# Epigenetic Alterations in PAH-Induced Childhood Asthma: An Intervention Using Sulforaphane

**DOI:** 10.3390/toxics13100809

**Published:** 2025-09-23

**Authors:** Xinyao Jiang, Xinfeng Xu, Jinyan Hui, Yuling Bao, Shuyuan Cao, Qian Wu

**Affiliations:** 1 China International Cooperation Center (CCC) for Environment and Human Health, School of Public Health, Nanjing Medical University, Nanjing 211166, China; jiang_xy0604@stu.njmu.edu.cn (X.J.); scottsmith@stu.njmu.edu.cn (X.X.); 2024120805@stu.njmu.edu.cn (J.H.); caoshuyuan@njmu.edu.cn (S.C.); 2 Department of Health Inspection and Quarantine, School of Public Health, Nanjing Medical University, Nanjing 211166, China; 3 Department of Respiratory, Children’s Hospital of Nanjing Medical University, Nanjing 210008, China; baoyuling@aliyun.com

**Keywords:** PAH exposure, childhood asthma, sulforaphane, epigenetic modification

## Abstract

DNA methylation holds promise for the early detection of tissue damage, making it crucial for identifying polycyclic aromatic hydrocarbon (PAH)-associated epigenetic biomarkers in childhood asthma. Sulforaphane (SFN), as a potential epigenetic modulator, can alleviate the adverse effects of environmental pollutants. This study quantified serum PAHs in 370 children via gas chromatography–mass spectrometry, assessed the methylation of target genes using bisulfite sequencing PCR (BSP), and performed mediation analysis to estimate the mediating effects of methylation levels between PAHs and childhood asthma. Murine models exposed to PAHs prenatally or postnatally, with offspring challenged with ovalbumin (OVA), were analyzed for lung DNA methylation. In vitro, HBE cells and HBSMCs treated with benzo(a)pyrene (BaP) and/or SFN were tested for inflammatory cytokines, methylation-related enzymes, and matrix metallopeptidase 9 (*MMP9*) modifications. The results showed total PAHs were associated with childhood asthma, with mediating effects of long interspersed nuclear element-1 (*LINE-1*) methylation. Prenatal PAH exposure enriched differentially methylated genes in the extracellular matrix (ECM)-receptor interaction pathway, while postnatal exposure enriched those in purine metabolism, and postnatal exposure also elevated *Mmp9* expression via hypomethylation. BaP increased the expression of interferon gamma (*IFN-γ*), interleukin-4 (*IL-4*), interleukin-17A (*IL-17A*), transforming growth factor beta 1 (*TGF-β*), and ten-eleven translocation methylcytosine dioxygenases (*TETs*), and it upregulated *MMP9* via enhancer hypomethylation and H3K27ac enrichment, while SFN reversed these effects by downregulating histone methyltransferase (HMT), leading to reduced H3K4me1 and subsequent H3K27ac depletion, thus suppressing *MMP9* transcription. This study demonstrates that DNA methylation mediates PAH–childhood asthma associations, with distinct patterns in different exposure windows; *MMP9* could serve as a crucial target for epigenetic modification during lung inflammation induced by PAH exposure, and SFN reverses PAH-induced epigenetic changes, aiding prevention strategies.

## 1. Introduction

Polycyclic aromatic hydrocarbons (PAHs) are a typical type of environmental chemical pollutant and are listed as typical persistent organic pollutants (POPs). PAHs are mainly derived from the incomplete combustion or thermal degradation of energy sources and other organic substances. After cooling in the air, most of them are adsorbed onto fine particulate matter (with a particle size of ≤10 µm) and can enter the human body through various pathways, such as respiration, diet, and skin contact [1]. In the past, the hazards of PAHs were mainly found in factories, such as coal, oil, coal tar mining, aluminum smelting, steel casting, and other industrial sites. Therefore, most of the research on the impact of PAHs on human health focused on their effects under special environments or occupational exposures [2,3,4]. With the acceleration of urbanization and the continuous increase in vehicular emissions, PAHs have become a critical air pollution issue. The health effect of PAHs has been extended from the occupationally exposed population to the general public. Birth cohort studies have shown that maternal exposure to PAHs derived from vehicle exhaust systems is a risk factor for asthma-related symptoms in offspring [5,6,7,8,9,10]. And repeated high polycyclic aromatic hydrocarbon exposures in children induce allergic sensitization [11,12,13].

Currently, the main mechanism of the diseases caused by PAHs is the DNA genetic damage they induce [14,15,16,17]. Primarily, after a series of enzymatic metabolic activations in the body, PAHs were metabolized into electrophilic active metabolites. These metabolites can form covalent bonds with the amino groups on the outer rings of deoxyadenine and deoxyguanine in DNA molecules, thereby forming PAH-DNA adducts and subsequently inducing gene mutations [18]. It has been reported that intrauterine exposure to PAHs induces epigenetic alterations in the spatial structure of genetic material, leading to DNA damage through the formation of PAH-DNA adducts [19].

Epigenetic alterations, such as DNA methylation and histone modifications, have been implicated as key candidates for mediating adaptive responses to environmental change [20]. The establishment of DNA methylation patterns during embryogenesis plays crucial roles in gene transcription and chromosome stability [20,21,22,23]. Additionally, ten–eleven translocation protein contributes to epigenetic reprogramming by mediating DNA demethylation, which is crucial for cellular differentiation and stem cell maintenance [24,25]. Our previous research identified an association between serum fluoranthene level and childhood asthma. Furthermore, this relationship was significantly mediated by DNA methylation in long interspersed nuclear element-1 (*LINE-1*) and H3K4me3 levels in the interleukin-17A (*IL-17A*) promoter region among school-age children [26]. It is likely that persistent alterations in children’s DNA methylation caused by environmental PAH exposure represent a potential mechanism for the development of chronic diseases.

Additionally, there is increasing evidence showing that phytochemicals become an effective regulatory factor for epigenetic markers, by directly influencing human epigenetic mechanisms [27,28]. Sulforaphane (SFN) is an isothiocyanate predominantly present in cruciferous vegetables, such as broccoli and mustard [29]. The antioxidant and anti-inflammatory effects of SFN are mainly attributed to its regulator role in nuclear factor erythroid 2-related factor 2, nuclear factor κB, and epigenetic modifications [30,31,32]. Juengel et al. found that SFN is one of the natural inhibitors of histone deacetylase (HDAC), which suppresses tumor progression by restoring the balance of acetylation levels through HDAC inhibition [33]. Moreover, SFN act as a potential regulator of DNA methyltransferase (DNMT). It was reported to regulate DNA methylation by dose-dependently inhibiting DNA methyltransferase 1 (*DNMT1*) and DNA methyltransferase 3A (*DNMT3A*). Sulforaphane can specifically act on the CpG of the first exon of human Telomerase Reverse Transcriptase, leading to demethylation. A chromatin immunoprecipitation assay analysis showed that sulforaphane increases the acetylation of chromosomal histones H3, H3K9, and H4 and reduces the trimethylation levels of H3K9 and H3K27 [34]. However, the influence of SFN on DNA methylation is still limited. Further research is required in order to address the anti-inflammatory mechanisms of SFN mediated through epigenetic modifications.

Overall, DNA methylation typically precedes genetic changes and represents an early-stage effect biomarker. The assessment of DNA methylation thereby allows for the early detection of biological damage. Consequently, screening candidate genes associated with PAH-exposure-related asthma and establishing them as epigenetic biomarkers hold promise for developing preventive strategies against childhood asthma induced by PAHs. Therefore, this study endeavors to clarify via in vitro and in vivo experiments that exposure to PAHs can exert an influence on the initiation and advancement of childhood asthma through epigenetic mechanisms, and whether or not SFN intervention can reverse the PAH-induced epigenetic changes.

## 2. Materials and Methods

### 2.1. Sample Collection

The Nanjing Medical University Clinical Research Ethics Committee, Nanjing, China, reviewed and approved the protocols of this study (Approval Number: 201902080-1). Written informed consent was obtained from the participants’ parents for the use of samples in this study. This study enrolled 185 physician-diagnosed asthmatic children from Nanjing Children’s Hospital (2021–2022) and 185 non-asthmatic controls without allergic diseases from Jiangsu Provincial Hospital of Chinese Medicine during the same period. The inclusion criteria were as follows: (1) the asthma group consisted of children with physician-confirmed asthma diagnosis [35]; (2) the control group consisted of children without physician-diagnosed asthma or allergic disorders; and (3) written informed consent was obtained from all parents/guardians with Helsinki Declaration compliance. The exclusion criteria were as follows: (1) immune system abnormalities, particularly immune-mediated disorders (e.g., allergic purpura); (2) vital organ dysfunction, involving cardiac, hepatic, or renal systems; and (3) recent acute infections or asthma exacerbations (within 3 months prior to sampling). Peripheral blood was collected and centrifuged (3000 rpm, 10 min, 4 °C). Serum and white blood cells were separated and stored at −80 °C for subsequent analysis. DNA was extracted following the protocol of the FastPure Blood DNA Isolation Mini Kit V2 (Vazyme, Nanjing, China).

### 2.2. Detection of Serum PAHs by Gas Chromatography–Mass Spectrometry

One hundred µL of the serum was mixed with 10 μL of a 100 ng/mL mixed internal standards solution, including Chrysene-d12, and Perylene-d12 (AccuStandard, New Haven, CT, USA). Then, 250 μL of 6 mol/L hydrochloric acid, 250 μL of isopropanol, and 1.5 mL extraction solution of n-hexane/methyl tertiary butyl ether (*v*/*v*, 1:1) were added. The mixture was vortexed for 2 min and allowed to stand until the layers were completely separated. The supernatant was collected. The above extraction procedure was repeated three times. The mixed extract was purified via solid-phase extraction via a 3 mL florisil cartridge (Anpel, Shanghai, China), and slowly eluted three times with 2 mL n-hexane: dichloromethane (*v*/*v*, 1:1) eluent in a 15 mL centrifuge tube. The mixed extract was evaporated with a nitrogen stream, redissolved in 100 μL of dichloromethane, and prepared for quantification.

The concentrations of the 10 PAHs defined as high-priority pollutants in the serum, including fluoranthene (Fla), pyrene (Pyr), benz(a)anthracene (BaA), chrysene (Chr), benzo(b)fluoranthene (BbF), benzo(k)fluoranthene (BkF), benzo(a)pyrene (BaP), indeno(l,2,3-cd)pyrene (INP), dibenz(a,h)anthracene (DBA), and benzo(g,h,i)perylene (BgP) (AccuStandard, New Haven, CT, USA), were measured via a gas chromatograph mass spectrometer (TRACE 1310, Thermo Fisher Scientific, Waltham, MA, USA) with a chromatographic column (DB-5MS, 30 m, liquid film thickness 0.25 μm, internal diameter 0.25 mm). The oven temperature was programmed as follows: initial temperature at 60 °C held for 1.5 min, followed by an increase to 100 °C at a rate of 8 °C/min (held for 1 min), then ramped up to 250 °C at 15 °C/min, and, finally, increased to 310 °C at 5 °C/min (held for 10 min). The flow rate of N_2_ was 10.0 L/min and the injection volume was 1 µL. The ionization mode was Electron Impact ionization (EI). The temperature of the transfer line was set to 280 °C. SRM mode for Electron Impact ionization (EI) was applied. If the concentration was below the limit of detection (LOD), it was reported as not detected (ND) and assigned a concentration of LOD/√2. The retention times, parent ions, daughter ions, and collision energies of the target compounds are shown in Table S1.

### 2.3. Animal Study

All experiments were approved by the Nanjing Medical University Institutional Animal Care and Use Committee (IACUC-11771). C57BL6 mice (six weeks old, 16.0 ± 2.0 g) were purchased from Nanjing Medical University Animal Center, Nanjing, China. All mice were housed under controlled conditions with a temperature of 22 ± 1 °C, relative humidity of 55 ± 5 %, and a 12/12 h light/dark cycle. Food and water were provided ad libitum. The mice were mated in cages with a 2:1 ratio of female to male after seven days. When the vaginal plug was detected, it was recorded as 0.5 days of gestation (G 0.5). Pregnant mice were randomly divided into four groups: control group, asthma group, prenatal PAH-exposed group (pre-PAHs group), and postnatal PAH-exposed group (post-PAHs group), with details provided in Supplementary Materials and Figure S1.

The PAH-exposed group received a 50 μg/kg dose of PAH mixture via intranasal administration (10 μL/nostril). The PAH mixture was produced by our lab according to the proportional distribution of atmospheric PAHs that was measured in our previous work [36] and the dosage was determined in accordance with the report of a Joint FAO/WHO Expert Committee [37]. Prenatal PAH exposure was conducted from G11–12 to G20–21 for a total of 10 days, while postnatal PAH exposure was performed from postnatal day (PND) 22 to day 26 for a total of 5 days.

An equal number of mice offspring were randomly selected from each group for subsequent experiments. The offspring in asthma group, prenatal PAH-exposed group, and postnatal PAH-exposed group were induced asthma by ovalbumin (OVA, Sigma-Aldrich, St. Louis, MO, USA) as follows: mice were sensitized by intraperitoneal injection of 2 mg/kg OVA mixed with 80 mg/kg aluminum hydroxide (InvivoGen, Toulouse, France) on PND29, PND36, and PND43. Seven days after the last sensitization, asthma was challenged by intranasal administration of 4 mg/kg OVA (12.5 μL) for three consecutive days.

All mice were sacrificed 24 h after the last OVA challenge. Blood samples were drawn from the orbit and placed at room temperature for 2 h before centrifugation to collect the serum. The serum was stored at −80 °C until further analysis. Tracheal puncture was performed, and 0.5 mL of 0.9% normal saline solution was instilled repeatedly twice. The fluid was then recovered by gentle aspiration and centrifuged to obtain the bronchoalveolar lavage fluid (BALF). Lung tissues were collected for histological analysis or frozen at −80 °C for subsequent experiments.

### 2.4. Histopathology

Lung tissues were collected and fixed in 10% neutral buffered formalin. After separating the lung lobes, tissues were cut into 2 mm^3^ blocks, embedded in paraffin, and sectioned into 3 μm-thick slices. Hematoxylin and eosin (H&E) staining was performed to observe lymphocyte infiltration in the respiratory tract from proximal to distal regions. Periodic Acid–Schiff (PAS) staining was conducted to assess the proliferation of goblet cells in the bronchial epithelium. Three mice from each experimental group were randomly selected for pathological analysis.

### 2.5. Immunity and Cytokines Analysis

The determination of total immunoglobulin E (IgE) and interleukin-4 (IL-4) concentrations in the serum or BALF of mice were performed using Mouse IgE Enzyme-linked immunosorbent assay (ELISA) Kit (AMEKO, Shanghai, China) and Mouse IL-4 ELISA Kit (Cusabio, Wuhan, China), respectively, according to the manufacturer’s instructions. White blood cell (WBC) count in the BALF was measured using the Hitachi 7100 automatic biochemical analyzer (Shanghai, China), followed by H&E staining and microscopic examination of cell smears.

### 2.6. Cell Culture

Human bronchial epithelial cells (HBE) and Human bronchial smooth muscle cells (HBSMCs) were cultured in DMEM (KeyGEN, Nanjing, China) containing 10% FBS (KeyGEN, Nanjing, China) supplemented with 1% penicillin–streptomycin (KeyGEN, Nanjing, China) at 37 °C with 5% CO_2_. Sulforaphane and benzo(a)pyrene (BaP, ≥96%, HPLC) were both purchased from Sigma-Aldrich (St. Louis, MO, USA). HBE were treated with BaP (0.1 nM), SFN (10 μM), and a combination of BaP and SFN for 4 days. HBSMCs were treated with BaP (0.1 nM), SFN (2 μM), and a combination of BaP and SFN for 4 days. Cells treated with dimethyl sulfoxide (DMSO) acted as vehicle control. The maximum concentration of DMSO in the culture medium was 0.1% (*v*/*v*).

### 2.7. Methyl-RAD

Genome-wide DNA methylation sequencing was performed using the methylation-dependent restriction enzyme sequencing method (Methyl-RAD). The sequencing and analysis were conducted by Shanghai OE Biotech Co., Ltd. (Shanghai, China). The genomic DNA isolated from mouse lung tissues was treated with FspEI enzyme for digestion, after which adaptors and sample-specific barcodes were added, and the fragments were amplified by PCR to generate the sequencing library. The library was then sequenced on the Illumina Novaseq PE150 platform. After sequencing, raw data underwent quality control steps, including the removal of low-quality reads and sequences containing excessive N bases. Clean reads were aligned to the reference genome using SOAP software (version 2.21) to identify differentially methylated sites (DMSs) and differentially methylated genes (DMGs). Functional enrichment analysis of DMGs, including Gene Ontology (GO) and Kyoto Encyclopedia of Genes and Genomes (KEGG) analyses, were both performed.

### 2.8. Bisulfite Sequencing PCR (BSP)

According to the manufacturer’s instructions, genomic DNA was extracted from WBC of subjects, frozen mouse lung tissues, and HBE cells and HBSMCs using TIANamp Genomic DNA kit (Tiangen, Beijing, China), and the concentration was measured using a nucleic acid protein analyzer (Thermo NanoDrop 2000, Wilmington, DE, USA). Two-hundred nanograms of genomic DNA was modified by using EZ DNA Methylation^TM^ Kit (Zymo Research, Irvine, CA, USA). Predict the 5′-CpG islands in the promoter regions of m-matrix metallopeptidase 9 (*Mmp9*), h-*LINE-1*, h-interferon-gamma (*IFN-γ*), h-interleukin-4 (*IL-4*), h-forkhead box P3 (*FoxP3*), and h-matrix metallopeptidase 9 (*MMP9*) genes, as well as in the enhancer regions of the h-*MMP9* gene by Methprimer (Version 2.0, the Li Lab.). The sequences of the polymerase chain reaction (PCR) primers used were as shown in Table S2. The amplification products were identified by 2.0% agarose gel electrophoresis and subsequently purified using the EZNA Gel Extraction Kit (Omega Bio-tek, Norcross, GA, USA).

Following the instructions provided in the pMD 19-T Vector Cloning Kit (Takara Bio Inc., Kusatsu, Shiga, Japan), the purified products were cloned into pMD 19-T Vector and then introduced into DH5α competent cells through heat shock transformation. Then, 50 μL of bacterial culture was inoculated onto LB-ampicillin plates and incubated overnight for blue–white screening. Eight clones from each sample were sequenced by General Biosystems (Anhui) Co., Ltd. (Nanjing, China). The data analysis was conducted by DNAMAN (Version 9, Lynnon Biosoft, Vandreuil, QC, Canada). Comparing the sequencing results of the samples with original gene sequence, methylation sites were identified. The percentage of methylation was calculated using the following formula: (number of methylated CG sites/(number of methylated CG sites + number of unmethylated CG sites)) × 100%.

### 2.9. Real-Time Quantitative PCR Analysis

According to the manufacturer’s instructions, total RNA was extracted from frozen mice lung tissues, HBE cells and HBSMCs using FreeZol Reagent (Vazyme, Nanjing, China), and the concentration was measured using a nucleic acid protein analyzer (Thermo NanoDrop 2000, Wilmington, DE, USA). Reverse transcription was performed using the HiScript II Q RT SuperMix for qPCR (Vazyme, Nanjing, China) following the instructions provided. The real-time fluorescent quantitative PCR solution was prepared using ChamQ Blue Universal SYBR qPCR Master Mix (Vazyme, Nanjing, China) according to the manufacturer’s instructions. The sequences for primers are listed in Table S3. For data analysis, *β-actin* and *GaPdh* were used as the reference gene for normalization in human cell samples and mouse samples, respectively, with measurements performed in triplicate. The relative expression levels of the target genes were calculated using the 2^(−ΔΔCt)^ method.

### 2.10. CUT&RUN-qPCR Analysis

The Hyperactive pG-MNase CUT&RUN Assay Kit for PCR/qPCR (Vazyme, Nanjing, China) was used to perform the cleavage under targets and release using nuclease (CUT&RUN) assay on cells. After chromatin digestion by Protein G-fused MNase, immunoprecipitation was performed using antibodies against H3K27ac (1:100, #8173, Cell Signaling Technology, Danvers, MA, USA) and H3K4me1 (1:50, #5326, Cell Signaling Technology, USA), with IgG (#3900, Cell Signaling Technology, Danvers, MA, USA) serving as a negative control. The released DNA was purified and quantified by qPCR. Primers were designed to amplify the enhancer region of *MMP9* gene (forward: 5′-ACCCTATGTACCGCTTCACTG-3′, and reverse: 5′-CTCACCATAGAGGTGCCGGA-3′). The relative binding levels of the target histone were calculated using the 2^(−ΔΔCt)^ method.

### 2.11. Statistical Analysis

Serum PAH levels were log-transformed to normalize the data and eliminate scale differences. A logistic regression model was employed to assess the relationship between total PAH concentration and childhood asthma. The mediating effect was calculated using Model 4 of the PROCESS macro for SPSS, with Hayes’ bootstrapping method employed to assess the mediating effect of DNA methylation on the association between PAH exposure and childhood asthma. All data have undergone tests for normality and homogeneity of variance (α = 0.05 for both). The Mann–Whitney U test, Student’s *t*-test, and Kruskal–Wallis test were used to calculate significance, respectively. SPSS 26.0 (IBM, New York, NY, USA) and GraphPad Prism 8.0.2 (GraphPad Software, San Diego, CA, USA) were used to perform the statistical analysis. * *p* < 0.05 was considered statistically significant.

## 3. Results

### 3.1. Interactions of PAH Exposure with DNA Methylation Increased the Risk of Childhood Asthma

**Figure 1:**
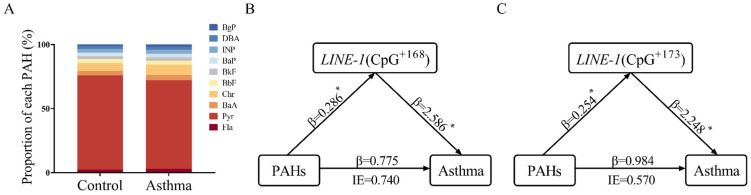
Proportions of each PAH in serum and the mediation effects of *LINE-1* methylation on the relationship between serum total PAHs and childhood asthma. (**A**) The proportion of each PAH in the control and asthma groups. (**B**,**C**) The mediating role of CpG^+168^ and CpG^+173^ of *LINE-1*. PAH, polycyclic aromatic hydrocarbon. *LINE-1*, long interspersed nuclear element-1. IE, indirect effect. β, effect size. * *p* < 0.05. The positions of CpG sites in *LINE-1* are annotated by base coordinates of the reference sequence (GenBank: X58075.1).

This study enrolled 185 asthmatic children, including 113 males and 72 females, with a mean age of 4.37 ± 2.57 years, and 185 control children, including 98 males and 87 females, with a mean age of 4.38 ± 2.53 years. The demographic data are available in Table S4**.** Owing to their greater potential for toxicity and bioaccumulation, this study primarily focuses on ten high-molecular-weight PAHs comprising four or more rings. The descriptive statistics of these PAHs are presented in Table S5. The proportions of these ten PAHs were similarly distributed in two groups, with Pyr exhibiting the highest proportion among them (Figure 1A). After adjusting for sex and age, a significant association was found between total PAHs and childhood asthma by logistic regression (OR = 2.558, *p* < 0.05).

The methylation status in the promoter regions of the *LINE-1*, *IFN-γ*, *IL-4*, *FoxP3,* and *MMP9* genes in the WBC isolated from subjects was examined. As shown in Table S6, the DNA methylation levels at multiple CpG sites of these target genes showed significant differences between the two groups and children with asthma exhibited higher DNA methylation levels (Figure S2). Then, the mediating effects of both site-specific CpG methylation rates and gene-wide mean methylation levels on the association between PAH exposure and childhood asthma were assessed. The analysis revealed CpG^+168^ and CpG^+173^ of *LINE-1* exhibited a significant mediating effect between total PAHs and childhood asthma (Figure 1B,C).

### 3.2. PAH Exposure Exacerbated Asthma Phenotypes

**Figure 2:**
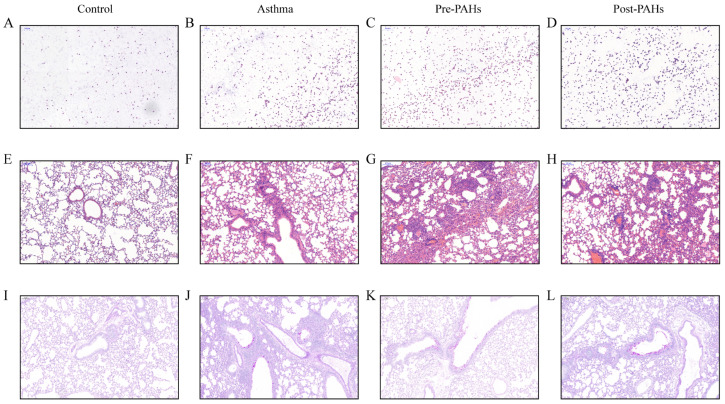
Histological changes in BALF and lung tissue. (**A**–**D**) Representative photomicrographs of inflammatory cell infiltration in BALF by H&E staining. (**E**–**H**) Representative photomicrographs of inflammatory cell infiltration in lung tissue by H&E staining. (**I**–**L**) Representative photomicrographs of mucus-secreting goblet cells in lung tissue by PAS staining. Scale bar, 100 μm; original magnification, ×10. BALF, Bronchoalveolar Lavage Fluid. H&E, Hematoxylin and Eosin. PAS, Periodic Acid–Schiff.

A histological observation indicated that PAH exposure aggravated asthma phenotypes. Significant inflammatory cell infiltration was observed in the BALF (Figure 2A–D) and lung tissue (Figure 2E–H) of both the asthma and PAH-exposed group. Additionally, a significant increase in mucus-secreting goblet cells was also demonstrated by PAS staining (Figure 2I–L). As shown in Table S7, the WBC counts, total serum IgE levels, and IL-4 concentrations in the BALF were elevated in the asthma group and PAH-exposed groups. Furthermore, the total serum IgE levels and IL-4 concentrations were significantly higher in the PAH-exposed asthma group than those in the asthma group.

### 3.3. PAH Exposure Induced Alterations in DNA Methylation Patterns in Offspring Lungs

**Figure 3:**
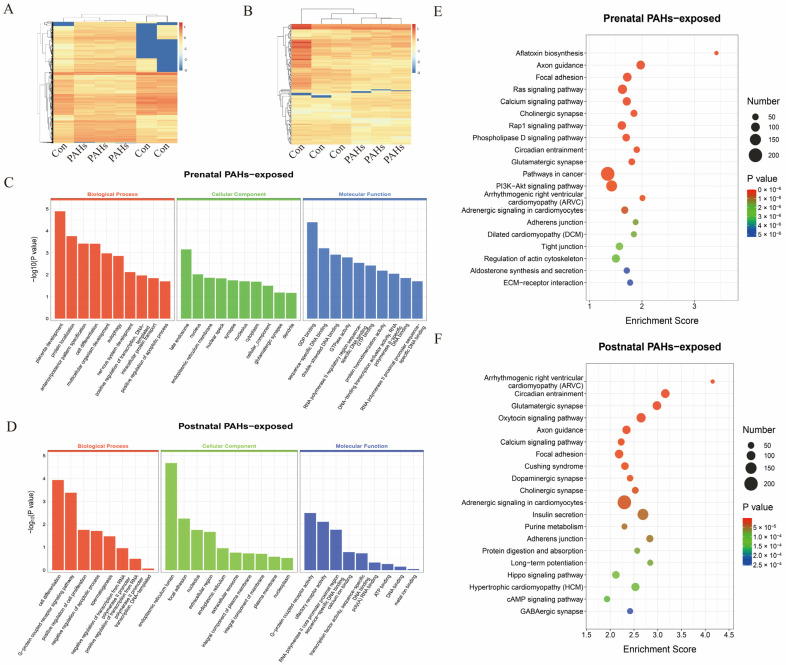
Genome-wide methylation profiling results based on Methyl-RAD technology. (**A**,**B**) Heatmap of differentially methylated sites in prenatal PAH-exposed group and postnatal PAH-exposed group, respectively, compared to the control. The smallest 1000 *p*-value were shown. (**C**,**D**) GO analysis of differentially methylated genes in prenatal PAH-exposed group and postnatal PAH-exposed group, respectively, compared to the control. GO, Gene Ontology. (**E**,**F**) The bubble plot of KEGG pathway analysis in prenatal PAH-exposed group and postnatal PAH-exposed group, respectively, compared to the control. The top 20 enriched pathways were shown. (*n* = 3). KEGG, Kyoto Encyclopedia of Genes and Genomes.

To investigate the epigenetic alterations induced by PAHs in lung tissues, a genome-wide methylation analysis was further performed using Methyl-RAD sequencing. Compared to the control group, the prenatal PAH-exposed group showed greater DNA methylation alterations than those in the postnatal PAH-exposed group, suggesting that the time-dependent PAH exposure played a critical role in shaping the offspring lung DNA methylation patterns (Figure 3A,B). Additionally, a GO analysis revealed that DMGs in the prenatal PAH-exposed group were significantly enriched in biological processes, such as placenta development, whereas DMGs in the postnatal PAH-exposed group showed enrichment in the cell differentiation process (Figure 3C,D). A KEGG pathway analysis revealed DMGs in the prenatal PAH-exposed group were enriched in the extracellular matrix (ECM)-receptor interaction pathway, whereas those genes were enriched in purine metabolism in the postnatal PAH-exposed group (Figure 3E,F).

**Figure 4:**
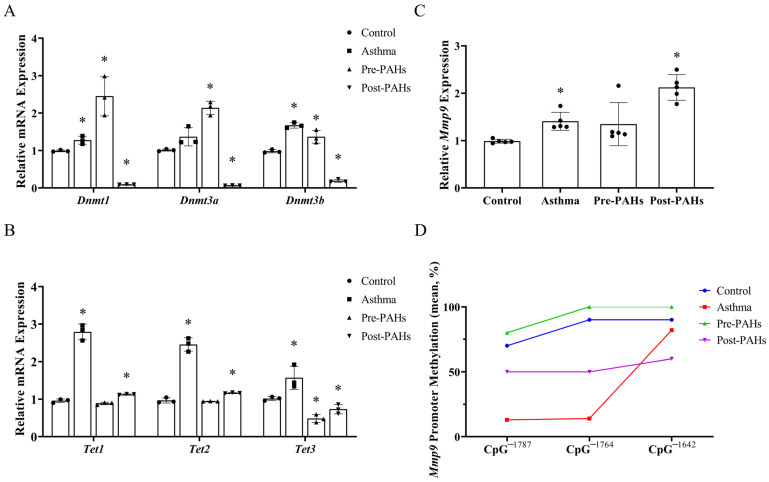
Different DNA methylation patterns induced by PAH exposure. (**A**,**B**) The expression of *Dnmt1*, *Dnmt3a*, and *Dnmt3b* (**A**) and *Tet1*, *Tet2*, and *Tet3* (**B**) in lung tissue. *Dnmt1*, DNA methyltransferase 1. *Dnmt3a*, DNA methyltransferase 3A. *Dnmt3b,* DNA methyltransferase 3B. *Tet1*, ten-eleven translocation 1. *Tet2*, ten-eleven translocation 2. *Tet3*, ten-eleven translocation 3. (**C**) The expression of *Mmp9* gene in lung tissue. *Mmp9*, matrix metallopeptidase 9. (**D**) The methylation level of lung tissue in *Mmp9* promotor region. The positions of CpG sites in *Mmp9* are mapped relative to its transcription start site (+/−). Data are presented as mean ± SD and analyzed by Student’s *t*-test. * *p* < 0.05, compared to the control.

The expression of methylation-related enzymes in offspring lung tissues was further examined. As shown in Figure 4A,B, following prenatal PAH exposure, the expression of DNA methyltransferases (DNA methyltransferase 1 (*Dnmt1*), DNA methyltransferase 3A (*Dnmt3a*), and DNA methyltransferase 3B (*Dnmt3b*)) was increased, whereas the *Tet3* expression was decreased. In contrast, postnatal exposure decreased the expression of DNA methyltransferases (*Dnmts*) and increased the expression of ten-eleven translocation 1(*Tet1*) and ten-eleven translocation 2 (*Tet2*), while the ten-eleven translocation 3 (*Tet3*) expression was decreased.

Genome-wide DNA methylation sequencing indicated hypermethylation at the *Mmp9* promoter and 5′ UTR region in the prenatal PAH-exposed group, whereas the postnatal PAH-exposed group showed no significant methylation changes in these regions relative to the control. Due to the key role of *Mmp9* in cancer pathways and the ECM-receptor interaction pathway, the *Mmp9* expression and methylation status in offspring lungs were examined. Compared to the control, the expression of *Mmp9* was significantly increased in both the asthma group and postnatal PAH-exposed group (Figure 4C). A BSP analysis of the *Mmp9* promotor region showed the corresponding hypomethylation in these two groups. In contrast, the prenatal PAH-exposed group exhibited hypermethylation, compared to the control (Figure 4D).

### 3.4. SFN Intervention Reverses the BaP-Induced Epigenetic Changes

BaP is widely recognized as the most toxic and is frequently employed as a representative indicator of overall PAH toxicity. Therefore, BaP was selected as a model PAH for treating HBE cells and HBSMCs in the in vitro experiment. In addition, to evaluate the potential of SFN in reversing BaP-induced epigenetic alterations, two treatment groups were designed: one receiving SFN alone (SFN group), and another receiving SFN in combination with BaP (Mix group).

BaP significantly increased inflammatory cytokine expression, such as *IFN-γ*, *IL-4*, *IL17-A,* and transforming growth factor beta 1 (*TGF-β*) in both HBE cells and HBSMCs. Furthermore, BaP exposure can differentially modulate the expression of *DNMT1*, *DNMT3A*, DNA methyltransferase 3B (*DNMT3B*), ten-eleven translocation 1 (*TET1*), ten-eleven translocation 2 (*TET2*), and ten-eleven translocation 3 (*TET3*) in both cell lines. Notably, following SFN intervention, the altered expression of the aforementioned DNA methylation regulators was partially reversed (Figure S3).

### 3.5. MMP9 DNA Methylation Acts as a Biomarker of BaP-Induced Epigenetic Changes

**Figure 5:**
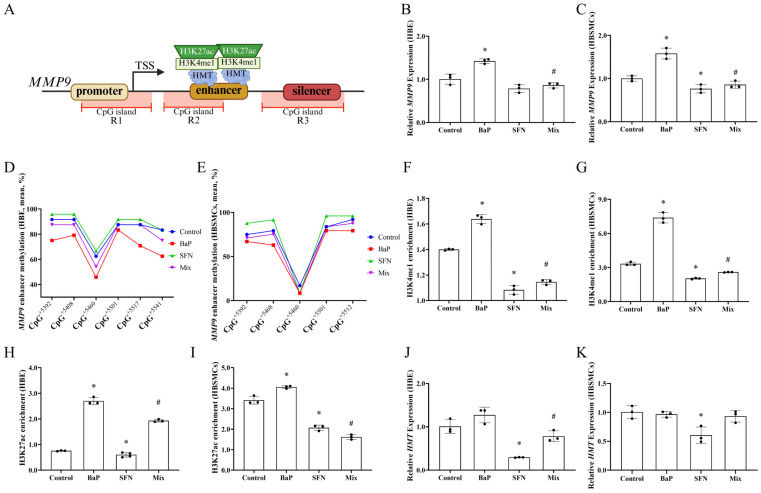
Epigenetic changes of *MMP9* triggered by BaP and SFN exposure. (**A**) Schematic diagram of in silico analysis for *MMP9* gene. The relative positions of CpG islands, promoters, enhancers, and silencers were identified based on National Center for Biotechnology Information (NCBI). *MMP9*, matrix metallopeptidase 9. TSS, Transcription start site. (**B**,**C**) The expression of *MMP9* in two cell lines. (**D**,**E**) The methylation level of different cell lines in *MMP9* enhancer region. The positions of CpG sites in *MMP9* are mapped relative to its transcription start sites (+/−). (**F**,**G**) H3K4me1 enrichment of different cell lines in *MMP9* enhancer region. (**H**,**I**) H3K27ac enrichment of different cell lines in *MMP9* enhancer region. (**J**,**K**) The expression of *HMT* in two cell lines. *HMT*, histone methyltransferase. Data are presented as mean ± SD and analyzed by Student’s *t*-test. * *p* < 0.05, compared to the control; # *p* < 0.05, compared to the BaP.

Although promoter methylation has been the primary research focus, recent evidence suggests that gene body methylation also significantly influences gene expression. Given the distinct time-dependent methylation patterns induced by PAH exposure in an animal study, an in silico analysis of the human *MMP9* gene was performed. As shown in Figure 5A, three key CpG islands in the gene body region were identified: R1 (46,010,329-46,011,438, 1110 bp), R2 (46,012,041-46,012,651, 611 bp), and R3 (46,013,604-46,014,607, 1004 bp).

The R2 region of *MMP9* contains an H3K4me1-marked enhancer that requires H3K27ac for transcriptional activation. As shown in Figure 5B,C, the *MMP9* expression was significantly increased by BaP treatment, accompanied by reduced methylation levels in the enhancer region (Figure 5D,E). The binding of H3K4me1 and the recruitment of H3K27ac were subsequently promoted by this epigenetic change, ultimately activating *MMP9* transcription (Figure 5F–I). Therefore, PAHs may contribute to asthma development and progression by modulating the *MMP9* expression through gene body methylation. However, SFN intervention reversed these effects by increasing enhancer region methylation and decreasing HMT activity, leading to less H3K4me1 binding and a subsequent decrease in H3K27ac recruitment, ultimately suppressing *MMP9* transcription (Figure 5B–K).

## 4. Discussion

Epidemiological studies have well-established a link between adverse environmental conditions and an increased risk of adult-onset diseases. While the underlying mechanisms are not fully understood, epigenetic dysregulation is widely hypothesized to be a key mediator in this phenomenon. Further elucidation of these mechanisms is crucial for understanding the long-term health implications of early life exposures to adverse environments. In this study, PAH exposure was associated with childhood asthma, with a mediating effect of *LINE-1* methylation. Moreover, prenatal and postnatal exposure to PAHs induced differential DNA methylation patterns in mouse lung tissues, with the former exhibiting a stronger influence on lung DNA methylation. While these effects were modest or mild, the results pointed to a plausible epigenetic mechanism for asthma pathogenesis triggered by this persistent organic pollutant. These results also extended our previous work in school-age children with asthma [26,36] and suggested an alteration in the DNA methylation level of asthma-related genes in the lung following air pollution exposure. A major strength of this study is the comprehensive assessment of PAH effects on DNA methylation across different developmental windows (prenatal vs. postnatal). Furthermore, it provides novel insights by linking PAH-induced epigenetic changes of *MMP9*—a key gene in extracellular matrix remodeling—to the pathogenesis of asthma. Finally, this study investigated the interventional potential of SFN against these PAH-induced epigenetic alterations.

Several reports have demonstrated a positive association between in utero/early-life PAH exposure and childhood asthma [38,39], which suggested that PAH exposure could exert toxicity by increasing inflammatory biomarkers and oxidative stress. Environmental exposure during these critical developmental windows not only is critical for immune system and airway development but can also induce epigenetic remodeling, such as alternations in DNA methylation. The metabolites of PAH have the capability to bind with DNA, resulting in the formation of DNA adducts. Notably, cytosines within CpG regions are frequently prone to adduct formation [40]. Several studies have evaluated DNA methylation in PAH-exposed human populations, both in vitro or in vivo. All these studies have reported different results in terms of DNA methylation status. Some of them demonstrated that PAH exposure is associated with hypomethylation. For example, Herbstman et al. found that prenatal exposure to PAHs was associated with global DNA hypomethylation in umbilical cord blood leukocytes [19]. Conversely, other studies have reported hypermethylation at various genetic loci following PAH exposure. For instance, the acyl-CoA synthetase long-chain family member 3 5′-CpG island(s) (5′-CGI) methylation status was positively and significantly associated with the maternal PAH exposure level and further associated with PAH-associated childhood asthma [6]. In addition, it was found in our previous study that the *LINE-1* methylation level had a significant mediation effect between serum Fla and childhood asthma [26]. In this study, although the effect of prenatal exposure to PAH on DNA methylation was modest, various genes associated with DNA methylation were significantly enriched in the biological processes related to placental development, as well as in cancer-associated pathways and the ECM-receptor interaction pathway. This aligns with the prior evidence that PAH can penetrate the placental barrier, thus posing potential adverse effects [41]. And postnatal exposure to PAH induced different changes in the DNA methylation of lung tissue compared with that of prenatal exposure to PAH. As demonstrated in our previous study, the differentially expressed metabolites in the urine metabolome of asthmatic children were involved in the purine metabolism pathways. Moreover, a mediation analysis revealed that 7-methylguanine mediated the association between urinary 1-hydroxypyrene (1-OHPyr, a PAH internal exposure biomarker) and childhood asthma [36]. These findings were further validated in animal experiments, which showed increased levels of 7-methylguanine in response to PAH exposure [26]. Given that 7-methylguanine is a valid indicator of the global DNA methylation status [42], these results suggested PAH exposure may alter epigenetic regulation. Environmental factors play a significant role in shaping DNA methylation profiles in utero, potentially leading to health outcomes in subsequent stages of life [43]. In addition, these contradictory results may arise from variables that influence the degree of DNA methylation—both globally and at specific genes—including endogenous factors (age, gender, and gene susceptibility) and exogenous factors (the time of exposure, the dosage of exposure, and the intake of folate as the donor of the methyl group) [44,45,46].

Recently, hydroxy methylation has become a new marker in epigenetic modulation. The 5-methylcytosine can be converted to 5-hydroxymethylcytosine by ten-eleven translocation (TET) family proteins [47], which plays an important role in the process of DNA demethylation and the regulation of gene expression. The pioneering work from Hong et al. demonstrated the importance of the TET-mediated pathway in asthma by revealing that traffic-related air pollution disrupts the *TET* expression and global DNA hydroxy methylation patterns, thus providing a mechanistic link between air pollution and asthmatic pathogenesis [23,48]. Reactive oxygen species (ROS) can be generated in the process of PAH metabolism, which can induce DNA hydroxy methylation [49]. In our study, PAH also influenced the *TET* gene expression in mouse lung tissue, and HBE cells and HBSMCs, respectively. It is therefore speculated that PAH can exhibit toxicity by the mechanism of DNA methylation/hydroxy methylation [50].

Based on genome-wide DNA methylation data, *MMP9* was selected for the analysis of its methylation status. This gene is a member of the matrix metalloproteinase family (MMPs), possessing a potent proteolytic capacity to degrade extracellular matrix components. MMPs play a critical role in physiological and pathological processes, including fibrosis, inflammation [51], and the remodeling of the extracellular matrix. The overexpression and dysregulation of *MMP9* is associated with various diseases. For example, BaP, the most well-known PAH, promoted the proliferation and metastasis of gastric cancer cells by upregulating *MMP9* via the aryl hydrocarbon receptor signaling pathway [52], of hepatoma cells via the p38 mitogen-activated protein kinase pathway [53], and of prostate carcinoma cells via the Janus kinase 2-signal transducer and activator of transcription 3 pathway [54]. A meta-analysis showed that *MMP9* polymorphisms was associated with asthma risk [55]. ORMDL sphingolipid biosynthesis regulator 3 promoted angiogenesis through upregulating vascular endothelial growth factor and *MMP9* in asthma airway remodeling. Thus, the regulation and inhibition of *MMP9* represent a crucial therapeutic target for various diseases. A more comprehensive understanding of the transcriptional regulation mechanism of the *MMP9* gene is required. Methylation in the promoter region of the *Mmp9* gene is an important mechanism for the regulation of *Mmp9* in mouse lymphoma [56]. In our study, postnatal PAH exposure in mice led to significant hypomethylation in the *Mmp9* promoter region and a significant increase in *Mmp9* gene expression in the lung. In contrast, BaP treatment in cell lines affected DNA methylation within the intragenic region. DNA methylation not only exists in the promoter region, but also in the intragenic or intergenic region [57]. Kulis et al. found that methylated gene-body CpG was positively or negatively associated with expression in the absence of DNA methylation changes in promoter regions. In addition, histone modifications are increasingly recognized for their fundamental role in the pathogenesis of inflammatory diseases like asthma, such as their ability to regulate chromatin accessibility and determine the functional state of enhancers and promoters, thereby influencing the expression of key genes [23,48,58,59,60]. The human *MMP9* gene has three obvious CpG islands in the gene body in the in silico analysis. The R2 region of the *MMP9* gene contains a distal poised enhancer marked by H3K4me1 that sustains transcription. This enhancer was unable to activate the promoter due to the presence of only H3K4me1 and the absence of H3K27ac. In addition, in an abnormal micro-environment, H3K4me1 induced aberrant DNA hypermethylation to inhibit the enrichment of H3K4me3 and then inactivate transcription in cancer [61]. In this study, BaP induced the DNA methylation level in the enhancer in the gene body region, and recruited the binding of H3K27ac, thus activating the *MMP9* gene transcription. The R3 region of the *MMP9* gene has a silencer. Aberrant DNA methylation can induce the silencer to alter their silencing effects or convert into enhancer [62]. In this region, BaP treatment did not influence the DNA methylation status in our study.

The result of this study demonstrated that epigenetic mechanisms play an important role in mediating the detrimental effects of environmental exposure. However, positive environmental influences (e.g., dietary interventions) can also promote health by inducing protective epigenetic modifications [63,64]. SFN has been proposed to be chemopreventive agents to inhibit the development of cancer, exhibiting the characteristics of anti-oxidative stress and anti-inflammation [65]. Li et al. found that SFN suppressed the nicotine-induced expression of *MMP9* via inhibiting ROS-mediated AP-1 and NF-κB signaling in human gastric cancer cells [66]. It has been reported that SFN can modify the activities of DNMTs and HDACs in cancer cells [67,68]. Given that SFN is well-recognized for its epigenetic modulation capabilities, we further investigated the mechanism by which SFN reverses the expression of the *MMP9* gene induced by BaP.

In our study, SFN reversed the BaP-induced epigenetic changes at the enhancer region, resulting in a decreased *MMP9* expression. Our data suggested that SFN increased the enhancer methylation level and reduced the activities of histone methyltransferase (HMT), leading to a reduction in both H3K4me1 and H3K27ac recruitment. These observations indicated that SFN is an HMT inhibitor, which is also supported by a previous study [68].

Our findings indicated that SFN may serve as an epigenetic modifier by reversing the BaP-induced alternations in DNA methylation and histone acetylation at the *MMP9* enhancer region. This modulation could ultimately influence critical signaling pathways associated with cellular processes such as inflammation and extracellular matrix remodeling in HBE cells. These findings supported the potential therapeutic utility of SFN in mitigating the adverse effects of environmental toxins on lung health.

This study has several limitations. First, although white blood cell DNA can serve as a global epigenetic indicator, the organ-specific information it provides is limited. Second, despite the efforts to standardize the sampling procedures, the use of mixed murine lung tissue homogenates may have masked cell-type-specific epigenetic responses (e.g., epithelial cells, alveolar macrophages, and fibroblasts). Finally, our in vitro experiments only focused on a single, highly representative PAH compound (BaP). In fact, human exposure typically occurs to complex mixtures of numerous PAHs and other atmospheric pollutants (e.g., particulate matter, heavy metals, and nitrogen oxides). These components can interact in an antagonistic, additive, or synergistic manner, potentially leading to epigenetic alternations and health effects that cannot be predicted from studying individual compounds. Therefore, future investigations should pay attention to organ-specific epigenetic analysis and more complex, environmentally relevant mixtures to elucidate the epigenetic consequences of environmental pollutant exposure.

In conclusion, this is the first study to demonstrate the different DNA methylation patterns induced by PAHs between prenatal and postnatal periods. Furthermore, PAH exposure had a more profound impact on shaping the epigenetic pattern during the prenatal window. Differential DNA methylated genes, such as *MMP9*, could serve as a crucial target for epigenetic modification during lung inflammation induced by PAH exposure. In addition, PAH exposure was found to impact epigenetic modification in both the promoter and gene body regions. Furthermore, we indicated that SFN might function as an epigenetic modifier to modulate the PAH-induced aberrant epigenetic changes in childhood-asthma-related genes. All in all, our findings help in improving our understanding of factors involved in childhood asthma.

## Data Availability

The data that support the findings of this study are available from the corresponding author upon reasonable request.

## Supplementary Materials

The following supporting information can be downloaded at: https://www.mdpi.com/article/10.3390/toxics13100809/s1, Figure S1: The flowchart of murine experiments; Figure S2: Methylation level in promoter regions of *LINE-1*, *IFN-γ*, *IL-4*, *FoxP3,* and *MMP9* in white blood cells; Figure S3: Epigenetic reversal effects induced by SFN exposure; Table S1: Retention times, precursor/product ions, and collision energies of target compounds; Table S2: Primers for BSP sequencing in this study; Table S3: Primers for real-time PCR in this study; Table S4: Demographic characteristics of all subjects; Table S5: Serum PAH concentrations of all the subjects in this study; Table S6: DNA methylation levels at different CpG sites of genes; Table S7: WBC counts, total serum IgE levels, and IL-4 concentrations in the BALF.

## Abbreviations

The following abbreviations are used in this manuscript:

PAHs	polycyclic aromatic hydrocarbons
SFN	sulforaphane
OVA	ovalbumin
BaP	benzo(a)pyrene
TETs	ten-eleven translocation methylcytosine dioxygenases
HMT	histone methyltransferase
POPs	persistent organic pollutants
HDAC	histone deacetylase
DNMT	DNA methyltransferase
Fla	fluoranthene
Pyr	pyrene
BaA	benz(a)anthracene
Chr	chrysene
BbF	benzo(b)fluoranthene
BkF	benzo(k)fluoranthene
INP	indeno(l,2,3-cd)pyrene
DBA	dibenz(a,h)anthracene
BgP	benzo(g,h,i)perylene
EI	Electron Impact ionization
LOD	limit of detection
ND	not detected
PND	postnatal day
BALF	bronchoalveolar lavage fluid
H&E	hematoxylin and eosin
PAS	Periodic Acid–Schiff
IgE	immunoglobulin E
IL-4	interleukin-4
ELISA	enzyme-linked immunosorbent assay
WBC	white blood cell
HBE	human bronchial epithelial cells
HBSMCs	human bronchial smooth muscle cells
DMSO	dimethyl sulfoxide
Methyl-RAD	methylation-dependent restriction enzyme sequencing method
DMSs	differentially methylated sites
DMGs	differentially methylated genes
GO	Gene Ontology
KEGG	Kyoto Encyclopedia of Genes and Genomes
BSP	Bisulfite Sequencing PCR
PCR	polymerase chain reaction
CUT&RUN	cleavage under targets and release using nuclease
OR	odds ratio
*Mmp9*	matrix metallopeptidase 9
*LINE-1*	long interspersed nuclear element-1
*IFN-γ*	interferon-gamma
*IL-4*	interleukin-4
*FoxP3*	forkhead box P3
*MMP9*	matrix metallopeptidase 9
*IL-17A*	interleukin-17A
ECM	extracellular matrix
*Dnmt1*	DNA methyltransferase 1
*Dnmt3a*	DNA methyltransferase 3A
*Dnmt3b*	DNA methyltransferase 3B
*Tet1*	ten-eleven translocation 1
*Tet2*	ten-eleven translocation 2
*Tet3*	ten-eleven translocation 3
*TGF-β*	transforming growth factor beta 1
*DNMT1*	DNA methyltransferase 1
*DNMT3A*	DNA methyltransferase 3A
*DNMT3B*	DNA methyltransferase 3B
*TET1*	ten-eleven translocation 1
*TET2*	ten-eleven translocation 2
*TET3*	ten-eleven translocation 3
CGI	CpG island
1-OHPyr	1-hydroxypyrene
ROS	reactive oxygen species
AP-1	activator protein-1
