# Supplementary material for: Epigenetic Alterations in PAH-Induced Childhood Asthma: An Intervention Using Sulforaphane
Source: Toxics. 2025 Sep 23;13(10):809. doi: 10.3390/toxics13100809 (PMC12568041; doi:10.3390/toxics13100809)
Supplement: Supplementary file 1 [file toxics-13-00809-s001.zip › toxics-3845710-supplementary.pdf]

Epigenetic Alterations in PAH-Induced Childhood Asthma: An Intervention Using Sulforaphane

Xinyao Jiang [1,2,†], Xinfeng Xu [1,2,†], Jinyan Hui [1,2], Yuling Bao [3], Shuyuan Cao [1,2] and Qian Wu [1,2,*]

[1] China International Cooperation Center (CCC) for Environment and Human Health, School of Public Health, Nanjing Medical University, Nanjing 211166, China

[2] Department of Health Inspection and Quarantine, School of Public Health, Nanjing Medical University, Nanjing 211166, China

[3] Department of Respiratory, Children's Hospital of Nanjing Medical University, Nanjing 210008, China

* Correspondence: wuqian@njmu.edu.cn

† These authors contributed equally to this work.

The Scheme of Animal Experiment:

Ⅰ. Ethical Statement:

All experimental procedures were approved by the Nanjing Medical University Institutional Animal Care and Use Committee (IACUC-11771). All efforts were made to minimize animal suffering.

Ⅱ. Animals and Housing:

Strain and Age: C57BL/6 mice (six weeks old, 16.0 ± 2.0 g) were purchased from Nanjing Medical University Animal Center, Nanjing, China.

Housing Conditions: All mice were housed under controlled conditions with a temperature of (22 ± 1) °C, relative humidity of (55 ± 5) %, and a 12/12-hour light/dark cycle. Food and water were pro-vided ad libitum. The mice were mated in cages with a 2:1 ratio of female to male after seven days. When the vaginal plug was detected, it was recorded as 0.5 days of gestation (G 0.5).

Ⅲ. Experimental Design and Groups:

The overall experiment consisted of two phases: a parental (F0) exposure phase and an offspring (F1) asthma induction phase. Pregnant mice (F0) were randomly divided into four groups. Their offspring (F1) were used for the subsequent asthma model.

Control Group (F1): Offspring from pregnant mice that received no PAHs exposure and were not subjected to the OVA-induced asthma protocol.

Asthma Group (F1): Offspring from pregnant mice that received no PAHs exposure but were later subjected to the OVA-induced asthma protocol.

Prenatal PAHs-exposed Group (Pre-PAHs, F1): Offspring from pregnant mice that received PAHs exposure during gestation. These offspring were later subjected to the OVA-induced asthma protocol.

Postnatal PAHs-exposed Group (Post-PAHs, F1): Offspring from pregnant mice that received no PAHs exposure. These offspring themselves received PAHs exposure post-weaning and were later subjected to the OVA-induced asthma protocol.

Ⅳ. Interventions:

1) PAHs Exposure:

Formula: The PAHs mixture was prepared in our laboratory according to the proportional distribution of atmospheric PAHs measured in our previous work. The dosage (50 μg/kg) was selected based on the report of a Joint FAO/WHO Expert Committee.

Route: Intranasal administration (10 μL/nostril, total 20 μL).

Timing:

Pre-PAHs Group (F0 Pregnant mice): From gestation day 11 to 20 (for a total of 10 days).

Post-PAHs Group (F1 Offspring): From postnatal day 22 to 26 (for a total of 5 days).

2) OVA-Induced Asthma Model (in F1 Offspring):

Sensitization: Mice were sensitized by intraperitoneal injection on postnatal day (PND) 29, PND 36, and PND 43 with a solution containing 2 mg/kg ovalbumin (OVA, Sigma-Aldrich®, USA) mixed with 80 mg/kg aluminum hydroxide (InvivoGen, France).

Challenge: Seven days after the last sensitization, mice were challenged with intranasal administration of 4 mg/kg OVA (i.n. 12.5 μL) for three consecutive days (PND 50-53). The control group received intranasal saline.

Ⅴ. Sample Collection (24 hours after the last OVA challenge):

1)Blood Serum: Blood samples were drawn from the orbit and placed at room temperature for 2h before centrifugation to collect the serum, which was stored at −80℃ for total serum IgE levels analysis.

2)Bronchoalveolar Lavage Fluid (BALF): Tracheal puncture was made, and 0.5 mL of 0.9 % normal saline solution was instilled repeatedly twice. The fluid was then recovered by gentle aspiration and centrifuged to obtain BALF. The supernatant was for IL-4 concentration analysis. The cell pellet was for Hematoxylin and eosin (H&E) staining.

3)Lung Tissues: Lung tissues were collected and processed in two ways:

Fixed in 10% neutral buffered formalin, embedded in paraffin, sectioned, and stained with Hematoxylin and Eosin (H&E) and Periodic Acid-Schiff (PAS) to assess inflammatory infiltration and goblet cell hyperplasia/mucus production, respectively.

Snap-frozen in liquid nitrogen and delivered to Shanghai OE Biotech Co., Ltd. on dry ice for Methyl-RAD sequencing analysis. The remaining snap-frozen tissue was stored at -80°C for future RNA and DNA extraction, intended for quantitative PCR (qPCR) and Bisulfite Sequencing PCR (BSP), respectively.

The Scheme of Cell Experiment:

Ⅰ. Cell Culture:

Human bronchial epithelial cells (HBE) and human bronchial smooth muscle cells (HBSMCs) were maintained in DMEM (KeyGEN, Nanjing, China) containing 10% FBS (KeyGEN, Nanjing, China) supplemented with 1% penicillin–streptomycin (KeyGEN, Nanjing, China). All cells were cultured at 37°C in a humidified atmosphere of 5% CO_2. Upon reaching 70-80% confluence, cells were seeded into appropriate multi-well plates.

Ⅱ. Reagents and Compounds:

Sulforaphane (SFN) was purchased from Sigma-Aldrich (St. Louis, MO, USA).

Benzo(a)pyrene (BaP, ≥96% purity, HPLC confirmed) was obtained from Sigma-Aldrich (St. Louis, MO, USA).

A stock solution of BaP was prepared in dimethyl sulfoxide (DMSO) at a concentration of 0.1 mM.

A stock solution of SFN was prepared in DMSO at a concentration of 50 mM.

All stock aliquots were stored at -20°C and protected from light. Working concentrations were prepared fresh by diluting in complete cell culture medium immediately before use. The final concentration of DMSO in all treatment media, including the vehicle control, did not exceed 0.1% (v/v).

Ⅲ. Experimental Groups and Intervention Timeline:

The experiment was conducted over a continuous 4-day (96-hour) treatment period. The treatment media were refreshed every 48 hours to ensure compound stability and minimize metabolite accumulation.

Vehicle Control: Culture medium containing 0.1% DMSO)

BaP Group: 0.1 nM BaP

SFN Group: 10 μM SFN for HBE; 2 μM for HBSMCs

Combination Group: BaP 0.1 nM + SFN 10 μM for HBE; BaP 0.1 nM + SFN 2 μM for HBSMCs

After the 4-day treatment period, cells were harvested for downstream analyses according to the following specific requirements:

1) For RNA extraction and qPCR analysis: Total RNA was extracted using FreeZol Reagent (Vazyme, Nanjing, China). Gene expression levels were analyzed by quantitative real-time PCR (qPCR).

2) For DNA extraction and DNA methylation analysis: Genomic DNA was extracted using TIANamp Genomic DNA kit (Tiangen, Beijing, China). The DNA was then subjected to bisulfite conversion, and the methylation status of *MMP9* enhancer region was analyzed by Bisulfite Sequencing PCR (BSP).

3) For CUT&RUN assay: Cells were collected and processed for the CUT&RUN experiment according to the standard protocol to investigate the recruitment of specific histone modifications (H3K4me1 and H3K27ac) at *MMP9* enhancer region.

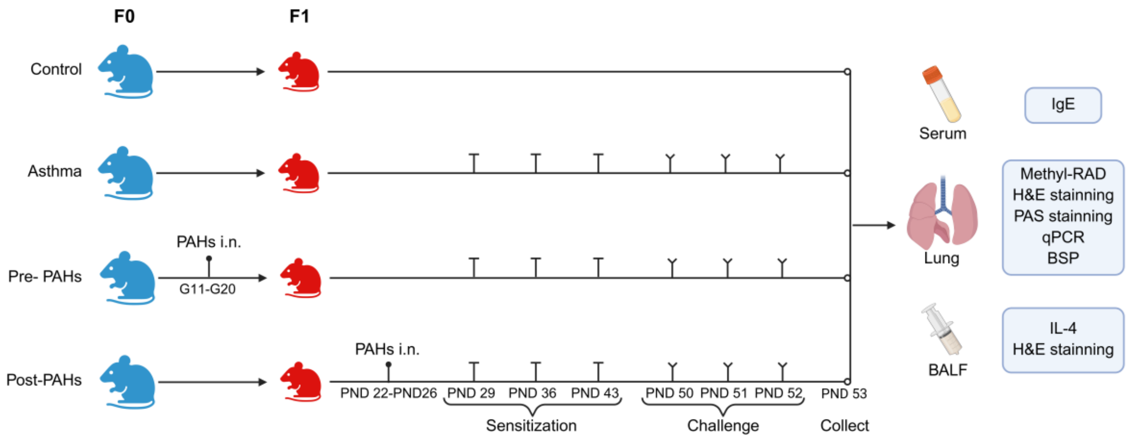

Figure S1 The flowchart of murine experiments

Schematic illustration of the animal experimental procedure. There were four groups: control group, asthma group, prenatal - polycyclic aromatic hydrocarbons (PAHs) - exposed (Pre - PAHs, PAHs administered to F0 during G11 - G20) group, and postnatal - polycyclic aromatic hydrocarbons - exposed (Post - PAHs, PAHs administered to F1 during PND 22 - PND 26) group. F0: pregnant mice; F1: offspring; PND: postnatal day; i.n.: intranasal administration.

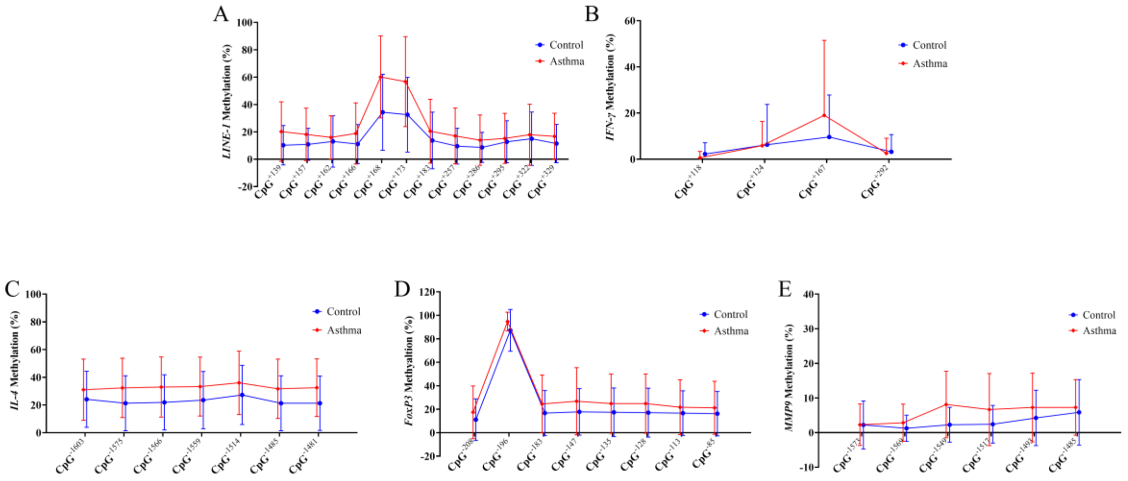

Figure S2 Methylation level in promoter regions of *LINE-1*, *IFN-γ*, *IL-4*, *FoxP3* and *MMP9* in white blood cells.

(A) The methylation level in the promoter regions of *LINE-1*. The positions of CpG sites in *LINE-1* are annotated by base coordinates of the reference sequence (GenBank: X58075.1). *LINE-1*, long interspersed nuclear element-1. (B) The methylation level in the promoter regions of *IFN-γ*. *IFN-γ*, interferon-gamma. (C) The methylation level in the promoter regions of *IL-4*. *IL-4*, interleukin-4. (D) The methylation level in the promoter regions of *FoxP3*. *FoxP3*, forkhead box P3. (E) The methylation level in the promoter regions of *MMP9*. *MMP9,* matrix metallopeptidase 9. The positions of CpG sites in *IFN-γ*, *IL-4*, *FoxP3* and *MMP9* are mapped relative to their transcription start sites (+/−). Data are presented as mean ± SD.

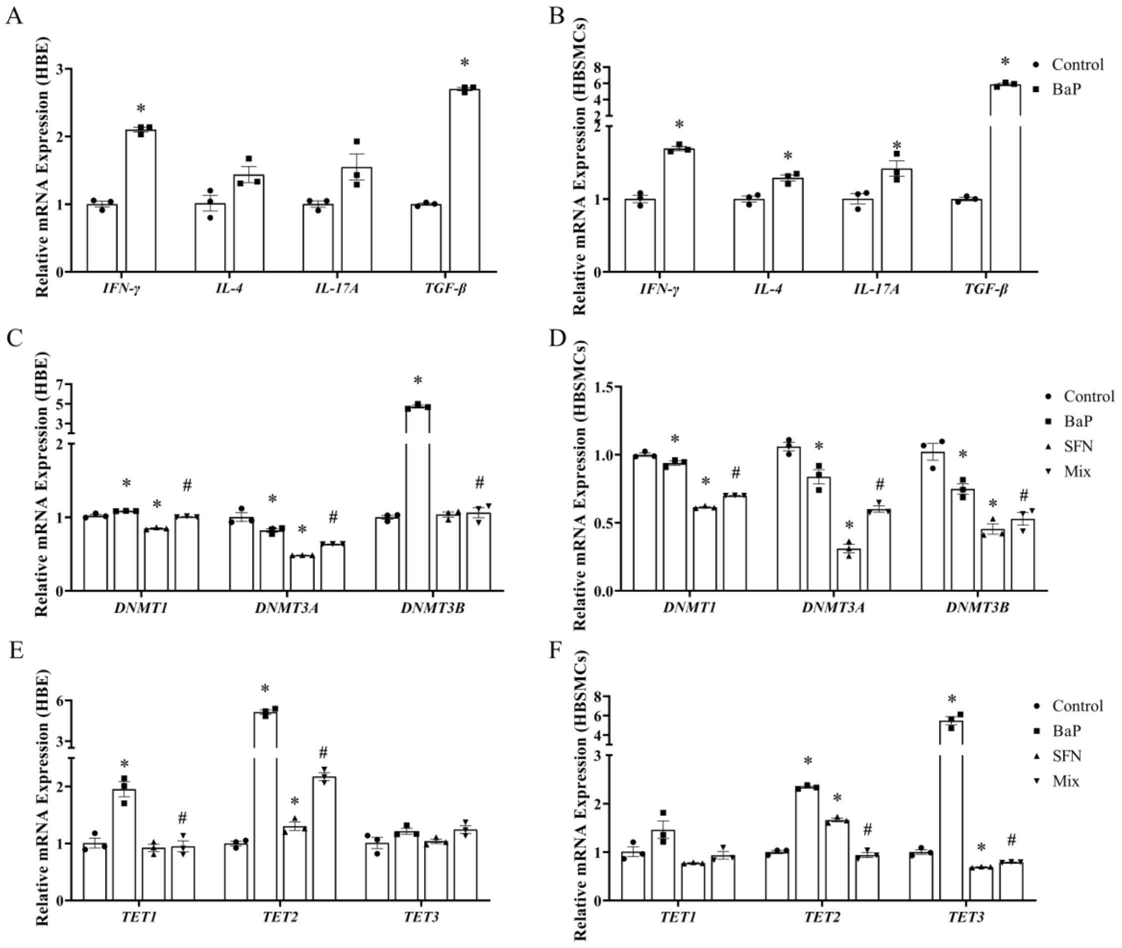

Figure S3 Epigenetic reversal effects induced by SFN exposure.

(A-B) The expression of inflammation gene (*IFN-γ*, *IL-4*, *IL-17A*, and *TGF-β*) in two cell lines. *IFN-γ*, interferon-gamma. *IL-4*, interleukin-4. *IL-17A*, interleukin-17A. *TGF-β,* transforming growth factor beta 1. (C-D) The expression of DNMTs (*DNMT1*, *DNMT3A* and *DNMT3B*) in two cell lines. DNMTs, DNA methyltransferases. *DNMT1,* DNA methyltransferase 1. *DNMT3A,* DNA methyltransferase 3A. *DNMT3B,* DNA methyltransferase 3B. (E-F) The expression of TETs (*TET1*, *TET2* and *TET3*) in two cell lines. TETs, ten-eleven translocation methylcytosine dioxygenases. *TET1*, ten-eleven translocation 1. *TET2*, ten-eleven translocation 2. *TET3*, ten-eleven translocation 3. * P<0.05, compared to the Control; $^{\#}$ P<0.05, compared to the BaP.

Table S1 Retention times, precursor/product ions, and collision energies of target compounds

PAHs	Retention times /min	Parent Ion m/z	Daughter Ion m/z	Collision Energies /V
Fla	18.34	202	200	30
Pyr	18.77	201.9	201.2	16
BaA	21.62	228.1	226	22
Chrysene-d12	21.65	240.2	236.2	30
Chr	21.72	228.1	202.1	25
BbF	24.83	252	250.2	26
BkF	24.92	252.1	250.2	32
BaP	25.83	252	250.1	30
Perylene-d12	26.01	264.2	260.2	35
INP	29.29	276	274.1	36
DBA	29.43	278.1	276.1	30
BgP	30.08	274.1	272.1	34

Table S2 Primers for BSP sequencing in this study

Genes	Forward (5′-3′)	Reverse (5′-3′)	Tm (℃)	Position	CpG site counts	Length (bp)
h-*LINE-1*	TTATTAGGGAGTGTTAGATAGTGGG	CCTCTAAACCAAATATAAAATATAATCTC	55	+113~+357	19	245
h-*IFN-γ*	GATTAGTTAAGTTTTTTGGATTTGTTAGT	ACAACCTATCAAAAATACTACAACA	55.3	+42~+321	4	279
h-*IL-4*	GGGAAGTGGAATAGAGGTAAAATTT	TAAAACTACAAACACCTACCACCAC	55.3	-1696~-1456	8	240
h-*FoxP3*	TGGTGAAGTGGATTGATAGAAAAGG	TATAAAAACCCCCCCCCACC	55.3	-230~+70	8	198
h-*MMP9*-promoter	GGGAGGTTAGGTGGGTAGATTATT	TCACTCTATCACCCAAACTAAAATACA	57.4	-1684~-1453	9	231
h-*MMP9*-enhancer (R2)	ATTGAGGGGTTTTTTTTGTATAAGG	CTAAACCCAAACTCTACTTCCAAAC	54.3	+5366~+5629	8	205
m-*Mmp9*-promoter	AGTGTTTTTTTATTAGATAAATTT	ATAAACTACTCTAACTCCTAAATCC	49.8	-1823~-1490	9	333

Note: The positions of CpG sites in h-*LINE-1* are annotated by base coordinates of the reference sequence (GenBank: X58075.1). The positions of CpG sites in h-*IFN-γ*, h-*IL-4*, h-*FoxP3*, h-*MMP9* and m-*Mmp9* are mapped relative to their transcription start sites (+/−). *LINE-1*, long interspersed nuclear element-1. *IFN-γ*, interferon-gamma. *IL-4*, interleukin-4. *FoxP3*, forkhead box P3. *MMP9*, matrix metallopeptidase 9. *Mmp9*, matrix metallopeptidase 9.

Table S3 Primers for real-time PCR in this study

Genes	Forward (5′-3′)	Reverse (5′-3′)
m-*GaPdh*	CAAGGTCATCCATGACAACTTTG	GGCCATCCACAGTCTTCTGG
m-*Dnmt1*	GTTCCCGCTGTTACCTCTTCC	CACCATCACGGCTCACTTCAC
m-*Dnmt3a*	GGAGGCGGTAGAACTCAAAGA	GGAAAGATCATGTACGTCGGG
m-*Dnmt3b*	ACTTTGCTGTCCTACTGAACTCC	TCAAGATTCCTGGCATGTAAC
m-*Tet1*	CATCTTCGGTCTGTGGGATGT	TGTTTGGTTCGTGAGCGTGTA
m-*Tet2*	GGCTATGTTGTCATTCTCCTTG	TGCCTTCACTACTAACTCCACC
m-*Tet3*	GCTGGTAGGGTTGCCATACTT	GAACGCCGTGATTGTTATCTT
m-*Mmp9*	CTGGACAGCCAGACACTAAAG	CTCGCGGCAAGTCTTCAGAG
h-*β-actin*	CATGTACGTTGCTATCCAGGC	CTCCTTAATGTCACGCACGAT
h-*IFN-γ*	QH03577S (Beyotime Biotechnology, Shanghai, China)	
h-*IL-4*	TTTGCTGCCTCCAAGAACA	TTGGCTTCCTTCACAGGACA
h-*IL-17A*	QH03769S (Beyotime Biotechnology, Shanghai, China)	
h-*TGF-β*	TACCTGAACCCGTGTTGCTC	CCGGTAGTGAACCCGTTGAT
h-*DNMT1*	GATCGAGACCACGGTTCCTC	CGGCCTCGTCATAACTCTCC
h-*DNMT3A*	GAGAACTGCAGGGCGAAGG	CGATGGCTCCACCTG GC
h-*DNMT3B*	CCAACAACACGCAACCAGTG	CGTCTTCGAGTCTTGTTCTCGTA
h-*TET1*	CCATATTATACACACCTTGGG	CTCCATGAACAGCCAAAAGAG
h-*TET2*	GGGCAGCCTTGTGGATGGCCC	GGAGCCCAGAGAGAGAAGGTT
h-*TET3*	ATGGACTCAGGGCCAGTGTAC	GTTCCCAGCCTCACGACTCATC
h-*MMP9*	TGTACCGCTATGGTTACACTCG	GGCAGGGACAGTTGCTTCT
h-*HMT*	GGCATCTTCCATGAGGAGCTT	ACAGCAATTCTGGTATCTTCCT

Note: *Dnmt1*, DNA methyltransferase 1. *Dnmt3a*, DNA methyltransferase 3A. *Dnmt3b*, DNA methyltransferase 3B. *Tet1*, ten-eleven translocation 1. *Tet2*, ten-eleven translocation 2. *Tet3*, ten-eleven translocation 3. *Mmp9*, matrix metallopeptidase 9. *IFN-γ*, interferon-gamma. *IL-4*, interleukin-4. *IL-17A*, interleukin-17A. *TGF-β*, transforming growth factor beta 1. *DNMT1*, DNA methyltransferase 1. *DNMT3A*, DNA methyltransferase 3A. *DNMT3B*, DNA methyltransferase 3B. *TET1*, ten-eleven translocation 1. *TET2*, ten-eleven translocation 2. *TET3*, ten-eleven translocation 3. *MMP9*, matrix metallopeptidase 9. *HMT*, histone methyltransferase.

Table S4 Demographic characteristics of all subjects

	ALL (N=370)	Control (N=185)	Asthma (N=185)	*p*
Age (years), Median (IQR)	4.0 (3.0, 5.0)	4.0 (3.0, 6.0)	4.0 (3.0, 5.0)	0.798
Sex, n (%)				0.141
Male	211 (57.03%)	98 (52.97%)	113 (61.08%)	
Female	159 (42.97%)	87 (47.03%)	72 (38.92%)	

Table S5 Serum PAH concentrations of all the subjects in this study

PAHs	Detection frequency (%)	Concentration Range (ng/mL)	Mean ± SD (ng/mL)	Median（ng/mL）	*P*
Fla	100	0.67-3.44	1.65±0.45	1.6	＜0.001
Pyr	100	9.37-251.74	47.05±31.55	43.47	0.459
BaA	100	1.27-9.00	2.54±0.74	2.44	＜0.001
Chr	100	1.85-19.05	4.58±1.75	4.08	＜0.001
BbF	99.8	ND-3.28	2.04±0.36	1.97	0.361
BkF	99.8	ND-2.97	1.61±0.55	1.26	0.003
BaP	99.8	ND-3.94	1.93±0.39	1.97	＜0.001
INP	94.3	ND-4.18	1.93±0.76	1.56	0.01
DBA	100	0.82-16.44	1.51±0.90	1.34	0.001
BgP	94.1	ND-11.49	1.32±0.64	1.33	0.006

ND: Not detected.

Table S6 DNA Methylation Levels at Different CpG Sites of Genes

Group	*LINE-1*			*IFN-γ*	*IL-4*				*FoxP3*		*MMP9*	
	**+139**	**+168**	**+173**	**+167**	**-1575**	**-1566**	**-1485**	**-1481**	**-196**	**-183**	**-1549**	**-1517**
Control	10	34	32	9.61	21.29	21.92	21.29	21.29	87.19	16.76	2.28	2.42
Asthma	20	60	57	18.99	32.32	32.99	31.7	32.5	94.69	24.51	8	7
P	0.024	0	0.001	0.462	0.022	0.029	0.032	0.02	0.11	0.227	0.003	0.045

Note: The positions of CpG sites in *LINE-1* are annotated by base coordinates of the reference sequence (GenBank: X58075.1). The positions of CpG sites in *IFN-γ*, *IL-4*, *FoxP3* and *MMP9* are mapped relative to their transcription start sites (+/−). *LINE-1*, long interspersed nuclear element-1. *IFN-γ*, interferon-gamma. *IL-4*, interleukin-4. *FoxP3*, forkhead box P3. *MMP9*, matrix metallopeptidase 9. Mmp9, matrix metallopeptidase 9.

Table S7 WBC counts, total serum IgE levels, and IL-4 concentrations in the BALF

**Group**	**White cells（×105/L）**	**IgE（μg/mL）**	**IL-4（pg/mL）**
Control	0.68±0.34	11.68±0.96	58.08±7.6
Asthma	2.51±0.91*	14.52±0.67*	128.34±10.2*
Prenatal PAHs-exposed	2.38±0.74*	17.46±1.00*#	213.45±11.0*#
Postnatal PAHs-exposed	2.46±0.87*	21.19±3.57*#	192.22±33.53*#

Note: * *P*<0.05, compared to the Control; # *P*<0.05, compared to the Asthma; n=3.
